# A Global View on Prevalence of Hypertension and Human Develop Index

**DOI:** 10.5334/aogh.2591

**Published:** 2020-06-29

**Authors:** Ziqian Zeng, Jiali Chen, Changfeng Xiao, Weizhong Chen

**Affiliations:** 1 Chengdu Medical College, CN

## Abstract

**Background:**

The incidence of hypertension has been increasing in the past decade. Little is known regarding the relationship between hypertension and human development index (HDI).

**Objectives:**

The objective is to identify the relationship between the prevalence of hypertension and human development index (HDI).

**Methods:**

An ecological study was conducted. The data from World Health Organization reports and United Nations Development Programme reports for 182 countries, including the HDI values, rates of tobacco use, physical inactivity, alcohol use, and salt intake. The Generalized Additive Models were implemented to assess the association between the prevalence of hypertension and the HDI.

**Results:**

Among 182 countries, the prevalence of hypertension ranged from 13% to 41%. The highest HDI value was 0.949 and the lowest was 0.352. In model 1, statistically significant associations were found in three populations, the largest R^2^ was 0.245. In model 2, the largest R^2^ was 0.485. In linear part, there was negative relationship in female population, while HDI was associated with prevalence of hypertension in all three populations, which was explained by spline function. The curve indicated that there were three intervals from low to high HDI. From 0 to 0.6 and 0.8 to 1, an evident decreasing trend of prevalence was found, while the rate increased when HDI was in the interval of 0.6 to 0.8.

**Conclusions:**

In this study, we identified the association between the prevalence of hypertension and the HDI and the underlying pattern of the relationship. The findings will aid the planning of hypertension control priorities and provide suggestions for interventions.

## Background

Hypertension, also known as high or raised blood pressure, is a condition in which the blood vessels have persistently raised pressure. It has been reported that one in four men and one in five women have raised blood pressure [1]. As a major modifiable risk factor for cardiovascular disease (CVD), it accounts for approximately 45% of global CVD morbidity and mortality [2,3,4,5]. Therefore, there are numerous studies to explore the genetic and environmental factors in order to prevent hypertension. However, few researches were conducted to discuss the association between human development index and hypertension.

Human development index (HDI) is universally used to assess the burden in countries in transition, which was suggested to be implacable for public health improvement and health resources allocation [6,7]. It is a summary measure of average achievement in three key dimensions of human development: living a long and healthy life, being knowledgeable, and having a decent standard of living. The health dimension is measured by life expectancy at birth. The education dimension is assessed by the mean of the number of years of schooling for adults aged 25 years and older and expected years of schooling for children of school-entering age. The standard of living dimension is represented by the gross national income per capita. Each of the three dimensions is calculated using the geometric mean of normalised indices.

Presently, in terms of environmental factors of hypertension, great attention was paid to individuals’ factors, such as ethnicity, education level, unhealthy behaviours, and metabolic diseases, et cetera [8,9]. Meanwhile, some studies have reported that societal and economic factors may be associated with the incidence and management of hypertension [10,11]. In addition, health condition, education level, and standard of living in a country also have a profound effect on the scale and profile of hypertension. These important indices for societal and economic status could be reflected by HDI. However, to date, there have been few studies exploring the association between the human development index (HDI) and hypertension.

In this study, we analyzed data including HDI, tobacco use, alcohol use, and physical inactivity to explore the underlying relationship between HDI and hypertension.

## Materials and Methods

### Data sources

The World Health Organization has compiled estimates of the worldwide prevalence of hypertension for 2015 by sex group for 182 countries [12]. In order to examine the prevalence of hypertension by levels of socioeconomic development, we linked the country-specific estimates for 2015 to the corresponding HDI scores for 2015. The HDI is estimated by the United Nations Development Programme (UNDP) and is an indicator of human capabilities within a country. For countries or regions with missing HDI scores, the data were not utilised in this analysis. In total, 182 countries were included in this study. As a result, the countries were classified into the HDI levels as shown in supplementary Table 1.

### Statistical analysis

Descriptive statistics, including the minimum, maximum, *P25, P75*, median, mean, and standard deviation, were used to describe the distribution of prevalence rates in 183 countries. Kruskal–Wallis *H* tests were used to test the median differences between the four HDI level countries; Spearman’s correlation coefficients between the HDI and prevalence then revealed the interrelationship. Firstly we used scatter plots to identify the data distribution. Secondly, we constructed a general additive model with tobacco use, physical inactivity, alcohol use, and salt intake as covariates to explore the patterns of their relationships between HDI and hypertension. We considered *P* values of less than 0.05 to be statistically significant. Statistical analyses were conducted using SAS, version 9.4.

## Results

In total, data from 182 countries were included in this study, the hypertension prevalence ranged from 13.0% to 41.0% and the HDI values were from 0.352 to 0.949. The distribution of tobacco use, alcohol use, physical inactivity and salt intake were presented in Table 1.

**Table 1:** Distribution descriptive for prevalence of hypertension and its related factors in 182 countries.


	N (missing)	Min	Max	Percentiles			Mean	SD

				25	50	75		

Prevalence (%)								
Male	182(0)	15.0	45.0	21.0	23.0	28.0	25.0	5.9
Female	182(0)	9.0	38.0	19.0	21.0	24.0	21.7	5.0
Total	182(0)	13.0	41.0	20.0	22.0	25.0	23.4	5.2
HDI	182(0)	0.352	0.949	0.579	0.733	0.811	0.700	0.153
Life expectancy at birth	182(0)	50.1	83.7	66.0	73.5	77.0	71.5	8.1
Expected years of schooling	182(0)	5.0	20.4	10.9	13.2	15.0	13.0	2.8
Mean years of schooling	182(0)	1.4	13.4	6.1	8.7	11.1	8.4	3.1
GNI per capita	182(0)	587.0	129916.0	3433.3	10492.5	23517.0	17072.3	18601.5
Alcohol (%)								
Male	180(2)	0.0	25.0	4.0	11.0	16.0	10.4	6.6
Female	180(2)	0.0	7.0	1.0	2.0	3.0	2.2	1.8
Total	180(2)	0.0	15.0	2.3	6.0	9.0	6.2	4.0
Physical inactivity (%)								
Male	155(27)	4.0	60.0	16.0	24.0	32.0	23.7	10.4
Female	155(27)	6.0	73.0	23.0	33.0	42.0	32.7	13.2
Total	155(27)	5.0	65.0	19.0	29.0	37.0	28.2	11.4
Salt/sodium intake (%)								
Male	180(2)	4.0	16.0	7.0	9.0	11.0	9.2	2.5
Female	180(2)	4.0	14.0	7.0	8.5	10.0	8.4	2.2
Total	180(2)	4.0	15.0	7.0	9.0	10.0	8.8	2.3
Tobacco use (%)								
Male	143(39)	1.0	77.0	21.0	30.0	41.0	32.1	14.0
Female	144(38)	0.0	44.0	2.0	6.0	18.0	9.8	9.5
Total	142(40)	4.0	47.0	14.0	21.0	27.0	21.2	9.1


### Correlations between hypertension and HDI, its components and other factors

As shown in Table 2, positive correlations between prevalence and HDI were reported in male and total population (*P* < 0.05), and the spearman’s estimates were 0.385 and 0.215 respectively, but there was no significant statistical trend found in female population (*P* > 0.05).

**Table 2:** The Spearman’s correlations analysis between prevalence (%) and HDI in different populations.


Factor	Spearman’s rho	Male	Female	Total

HDI	Correlation Coefficient	0.385**	–0.017	0.215**
	*P*	<0.001	0.815	0.004
	*N*	182	182	182
Life expectancy at birth	Correlation Coefficient	0.314**	–0.077	0.150*
	*P*	<0.001	0.299	0.043
	N	182	182	182
Expected years of schooling	Correlation Coefficient	0.406**	0.065	0.262**
	*P*	<0.001	0.385	<0.001
	N	182	182	182
Mean years of schooling	Correlation Coefficient	0.414**	0.062	0.262**
	*P*	<0.001	0.403	<0.001
	N	182	182	182
GNI per capita	Correlation Coefficient	0.319**	–0.078	0.152*
	*P*	<0.001	0.296	0.041
	N	182	182	182
Alcohol rate	Correlation Coefficient	0.517**	0.313**	0.456**
	*P*	<0.001	0.000	<0.001
	N	180	180	180
Physical inactivity rate	Correlation Coefficient	0.262**	–0.106	0.086
	*P*	0.001	0.189	0.290
	N	155	155	155
Salt/sodium intake rate	Correlation Coefficient	0.273**	0.177*	0.223**
	*P*	<0.001	0.018	0.003
	N	180	180	180
Tobacco use rate	Correlation Coefficient	0.254**	0.251**	0.371**
	*P*	0.002	0.002	<0.001
	N	143	144	142


*Note*: ** Correlation is significant at the 0.01 level (2-tailed). * Correlation is significant at the 0.05 level (2-tailed).

### Modeling differences over the HDI values

**Figure 1:**
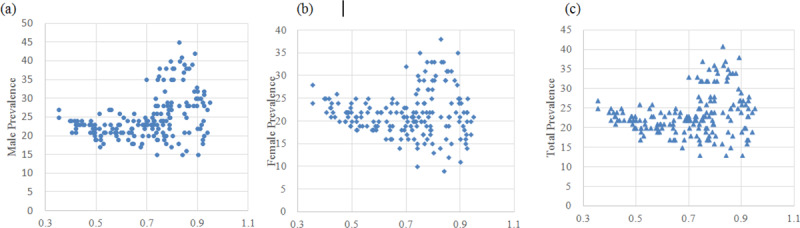
Scatter plots of HDI and prevalence in male, female, and total population.

**Figure 2:**
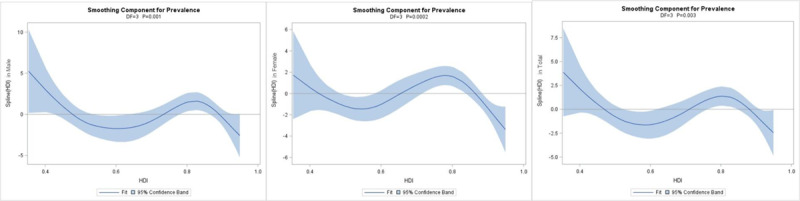
Smoothing components of spline HDI and prevalence in male, female, and total population in model 2.

Based on the scatter plots (Figure 1), a non-linear relationship exists between HID and hypertension. The GAM was conducted to explore the underlying pattern. The parts of spline in three populations were shown to be significant in model 1. The largest R square was found in the male population (R^2^ = 0.245). Further, the covariates, including tobacco use, alcohol use, physical inactivity, and salt intake, were included in model 2. Increased R squares were found in model 2, and significant association was found in the female population under linear function (*P* < 0.0001), while positive relationships were found in all groups under spline function (see Table 3). Fitness was improved and the relationship could be largely explained by models using natural cubic smoothing splines. The curve indicated that there were three trends from low HDI to high HDI. From 0 to 0.6 and 0.8 to 1, an evident decreasing trend of prevalence was found, while the rate increased when HDI was in the interval of 0.6 to 0.8 (Figure 2).

**Table 3:** Estimates from general additive models among three populations.


Source	Male		Female		Total	

	Parameter Estimate	*P*	Parameter Estimate	*P*	Parameter Estimate	*P*

**Model 1**						
Intercept	14.590	<0.0001	21.194	<0.0001	18.155	<0.0001
Linear(HDI)	14.838	<0.0001	0.689	0.7681	7.501	0.0019
Spline(HDI)	1.000	<0.0001	1.000	<0.0001	1.000	0.0002
**R Square**	0.245		0.095		0.148	
**Model 2**						
Intercept	13.180	<0.0001	25.946	<0.0001	18.446	<0.0001
Alcohol rate	0.482	<0.0001	1.251	<0.0001	0.689	<0.0001
Physical inactivity rate	–.002	0.9749	–0.030	0.3512	–0.015	0.7180
Salt/sodium intake rate	0.107	0.6006	0.730	0.0001	0.253	0.1879
Tobacco use rate	0.078	0.0150	0.248	<0.0001	0.167	0.0004
Linear(HDI)	4.831	0.2627	–20.084	<0.0001	–6.118	0.1321
Spline(HDI)	1.000	0.0010	1.000	0.0002	1.000	0.0030
**R Square**	0.474		0.485		0.428	


## Discussion

The increasing burden of hypertension seems to be associated with increased life expectancy and lifestyle factors. The HDI is found to be a broad marker of the scale and profile of hypertension, with the prevalence changing in magnitude according to increments in different HDI. Our analyses provide novel clues to the association between HDI and hypertension.

According to the descriptive characteristics, around 67% of countries with a prevalence of hypertension greater than 30% had a very high HDI. For example, Croatia, with a very high HDI level of 0.827, had the highest prevalence of 41%. This is consistent with a previous study, which also reported a high incidence in Croatia [13]. With an etiologic link between environmental factors and hypertension, the high prevalence may be attributed to unhealthy behavioural risk factors, such as smoking, unhealthy diet, harmful use of alcohol, lack of physical activity, excess weight, exposure to persistent stress, and so on [7,14,15,16]. The epidemiological evidence showed that some countries which had both high prevalence of hypertension and HDI level also had high physical inactivity rate or tobacco smoking rate. In turn, some countries with a low prevalence of hypertension and HDI level, such as Uganda and Zimbabwe, had a low physical inactivity rate, low salt intake, and low tobacco smoking rate [12]. As a result, considering these factors, we hypothesized that there might be an underlying pattern existing between prevalence of hypertension and HDI.

In this study, we conducted the general additive models to find the pattern. In the first model, we included the HDI as the only independent variable. The fitness of the model was not satisfied. Physical inactivity, tobacco use, alcohol use, and salt intake were then considered in our models. The R square was improved at this time. We found that there was not a simple linear relationship between prevalence and HDI. Results from natural cubic smoothing spines indicated in total population, from low to high HDI, the prevalence of hypertension had an increase in the interval of around 0.6 to 0.8. Using the predefined fixed cut-off values, the HDI levels were defined by UNDP as low (HDI < 0.55), medium (0.55 < HDI < 0.70), high (0.70 < HDI < 0.80), and very high (0.80 < HDI < 1.00). It implied that the prevalence of hypertension climbed up when the levels turned from medium to high. In these countries, higher HDI does not bring lower prevalence of hypertension, and more attention should be paid to discussing the possible reasons.

Even though this classification might not be deterministic and some countries are rapidly transiting from lower to higher levels of HDI, the global populations were classified by broad levels of HDI [17]. We conducted an ecological study to uncover the possible relationship between prevalence of hypertension and HDI. The novel findings will not only advance the understanding of etiology of this disease, but also provide a new insight into the prevention. Further research is still needed to investigate hypertension and other UNDP human development measures [18].

## Limitations

We collected the prevalence of hypertension, the rates of physical inactivity, tobacco use, alcohol use, and salt intake from World Health Organization reports. Even though the data has a standard calculation strategy and large coverage, the reporting system may vary in each country. Reassessment of the relationship would be conducted if more accurate data were available.

## Conclusion

A novel exposition of the effects on hypertension using HDI data and other unhealthy behaviour data was provided in this study. The trend was not consistent and a curve was found between the HDI and prevalence of hypertension in each population. An increasing trend indicated that the health practitioners should pay attention to those countries with both a higher prevalence and HDI values.
